# Integrating causal pathway diagrams into practice facilitation to address colorectal cancer screening disparities in primary care

**DOI:** 10.1186/s12913-024-11471-5

**Published:** 2024-08-30

**Authors:** Brooke Ike, Ashley Johnson, Rosemary Meza, Allison Cole

**Affiliations:** 1 Department of Family Medicine, University of Washington, Box 354982, Seattle, WA 98195-4982 USA; 2 Kaiser Permanente Washington, Health Research Institute, 1730 Minor Ave, Suite 1360, Seattle, WA 98101-1466 USA

**Keywords:** Colorectal cancer screening, Practice facilitation, Causal pathway diagram, Implementation, Screening disparities, Primary care, Quality improvement

## Abstract

**Background:**

Colorectal cancer (CRC) is the second leading cause of cancer death and the second most common cancer diagnosis among the Hispanic population in the United States. However, CRC screening prevalence remains lower among Hispanic adults than among non-Hispanic white adults. To reduce CRC screening disparities, efforts to implement CRC screening evidence-based interventions in primary care organizations (PCOs) must consider their potential effect on existing screening disparities. More research is needed to understand how to leverage existing implementation science methodologies to improve health disparities. The Coaching to Improve Colorectal Cancer Screening Equity (CoachIQ) pilot study explores whether integrating two implementation science tools, Causal Pathway Diagrams and practice facilitation, is a feasible and effective way to address CRC screening disparities among Hispanic patients.

**Methods:**

We used a quasi-experimental, mixed methods design to evaluate feasibility and assess initial signals of effectiveness of the CoachIQ approach. Three PCOs received coaching from CoachIQ practice facilitators over a 12-month period. Three non-equivalent comparison group PCOs received coaching during the same period as participants in a state quality improvement program. We conducted descriptive analyses of screening rates and coaching activities.

**Results:**

The CoachIQ practice facilitators discussed equity, facilitated prioritization of QI activities, and reviewed CRC screening disparities during a higher proportion of coaching encounters than the comparison group practice facilitator. While the mean overall CRC screening rate in the comparison PCOs increased from 34 to 41%, the mean CRC screening rate for Hispanic patients did not increase from 30%. In contrast, the mean overall CRC screening rate at the CoachIQ PCOs increased from 41 to 44%, and the mean CRC screening rate for Hispanic patients increased from 35 to 39%.

**Conclusions:**

The CoachIQ program merges two implementation science methodologies, practice facilitation and causal pathway diagrams, to help PCOs focus quality improvement efforts on improving CRC screening while also reducing screening disparities. Results from this pilot study demonstrate key differences between CoachIQ facilitation and standard facilitation, and point to the potential of the CoachIQ approach to decrease disparities in CRC screening.

**Supplementary Information:**

The online version contains supplementary material available at 10.1186/s12913-024-11471-5.

## Background

Colorectal cancer (CRC) is the second leading cause of cancer death and the second most common cancer diagnosis among the Hispanic population in the United States (US) [1]. The US Preventive Services Task Force recommends that adults age 45–75 screen for CRC as screening reduces CRC incidence and mortality [2–4]. However, CRC screening prevalence remains lower among Hispanic adults 45 years of age and older than among non-Hispanic white adults (64% vs. 74% in 2020) [5]. Primary care organizations (PCOs) have a range of evidence-based interventions (EBIs) to utilize for increasing CRC screening, including small media, clinician assessment and feedback, and patient reminders [6]. To reduce CRC screening disparities, it is imperative that efforts to implement CRC screening EBIs also consider their potential effect on existing screening disparities. Yet, there is no established approach for ensuring equity is integrated into implementation efforts in PCOs.

There have been recent calls to bring more of an equity focus to implementation science [7–9]. Brownson et al. suggest further examination of how to leverage existing implementation science methodologies to address equity determinants and improve health disparities [7]. Practice facilitation (PF) is an established implementation method for guiding PCOs in implementing EBIs [10–13]. PF draws on the Model for Improvement [14], which guides practice facilitators to ask three key questions: (1) What are we trying to accomplish? (2) How will we know that a change is an improvement? (3) What change can we make that will result in improvement? In order to select what changes to make, PF involves assessing existing systems, barriers to improvement, and potential interventions for improvement [14]. PF may be an approach to improving health equity, however, minimal research has been done on how or the degree to which PF may decrease health disparities [15].

A complementary implementation science visualization tool, the causal pathway diagram (CPD), provides a structure for implementers to be explicit about the outcomes they are trying to influence, barriers that are inhibiting those outcomes, and change strategies that may be poised to bring out improved outcomes [16]. By carefully articulating how strategies work, CPDs aim to improve their effectiveness [17, 18]. CPDs help implementers to consider whether a strategy will work under the local conditions by considering what is necessary for the strategy to work (i.e., preconditions) and what might enhance or diminish the effectiveness of the strategy (i.e., moderators). Within the context of PF, there could be potential in applying CPDs as a means to help facilitators ensure that the EBIs PCOs choose and the quality improvement (QI) strategies PCOs apply have genuine potential to address important local barriers to decreasing CRC screening disparities.

The Coaching to Improve Colorectal Cancer Screening Equity (CoachIQ) pilot study explores whether integrating CPDs into PF is a feasible and effective way to address disparities in CRC screening among Hispanic patients. The goal of this paper is to describe the CoachIQ practice facilitation approach and report changes in overall CRC screening rates and changes in CRC screening disparities before and after CoachIQ PF.

## Methods

### Study design

For our pilot study, we used a quasi-experimental, mixed methods design to evaluate feasibility and assess initial signals of effectiveness of the CoachIQ approach. Study procedures were reviewed by the University of Washington Human Subjects Division (STUDY00016086) and deemed to be human subjects research that qualifies for exempt status. Participants provided informed consent prior to participation.

### Study setting and recruitment

We partnered with the Washington, Wyoming, Alaska, Montana, and Idaho (WWAMI) region Practice and Research Network and the Washington Association for Community Health to recruit PCOs with Hispanic patient populations and CRC screening disparities. Of the eight PCOs approached directly about participating in CoachIQ, four had the capacity and interest to participate, and the study team selected the three PCOs with the larger Hispanic patient populations for inclusion. Coaching was provided at the organization level at three PCOs located in Wyoming (*n* = 1), Washington (*n* = 1), and Idaho (*n* = 1), and involved 4 practices. Two of the PCOs were federally qualified health centers and one was a hospital affiliated health center. We provided $1500 to each PCO to compensate them for time spent on research activities.

We worked with an organization that provides PF support to Washington state PCOs to improve CRC screening to identify and engage PCOs for the non-equivalent comparison group. Of the five PCOs approached to participate, three had the interest and capacity to share CRC screening data for the study. The three PCOs received coaching support during the same time period as the CoachIQ intervention. The three non-equivalent comparison group PCOs were federally qualified health centers and included 24 practices across three organizations providing care in Washington. Coaching support was provided at the organization level. We provided $500 to each comparison group PCO to compensate them for time spent on study-specific evaluation activities.

### Data collection

Throughout the study period (January 2023 – December 2023), the two CoachIQ practice facilitators kept field notes on their work with practices. At the end of each month, the two CoachIQ practice facilitators and the one comparison group practice facilitator completed a survey about coaching and QI activities. The survey was developed collaboratively with all three practice facilitators to include standard practice facilitator activities along with coaching elements related to CPD, such as whether the facilitator worked with the PCOs to understand how QI activities were expected to affect a prioritized barrier. The three practice facilitators received standardized instructions on how to interpret and respond to the survey questions. A copy of the survey is available in supplementary materials.

For each study site, we requested data on CRC screening rates from the electronic health records at two time points, the beginning and ending of coaching as was feasible for the participating practices. Data included CRC screening rates overall, among Hispanic patients, and among non-Hispanic patients. Additionally, we collected descriptive data about the participating PCOs and demographics information about their patient populations. Patient demographics data came from electronic health records data prior to the start of coaching (January 2024 for intervention practices, 2019 for comparison group practices).

### Data analysis

For our qualitative analysis, we used a basic qualitative descriptive approach [19] as the aim of the qualitative work was to identify and illustrate case examples of the CoachIQ approach as experienced by participating PCOs. A trained and experienced qualitative analyst (BI) independently reviewed and hand-coded practice facilitator field notes for examples of the facilitator and PCO applying CPDs to the QI work. The qualitative analyst created data displays of poignant examples of the application of the CoachIQ approach and reviewed these displays with the larger study team (AC, AJ, and RM) to reflect on their accurate representation of the data and experiences of practices.

For our quantitative analysis, we conducted descriptive analyses. We determined the baseline CRC screening disparity by calculating the difference between the non-Hispanic CRC screening rate and the Hispanic CRC screening rate. We compared baseline data with post-coaching data for the CoachIQ and comparison PF organizations. We also conducted descriptive analyses of participant PCO descriptive information and practice facilitator monthly coaching activity data.

### CoachIQ program

Each CoachIQ organization received approximately 12 months of QI support from two practice facilitators (one lead and one support) and a clinical advisor, who were also members of the study team. The CoachIQ organizations had no prior coaching on improving CRC screening. CoachIQ practice facilitators had 8 years (BI) and 3 years (AJ) of prior coaching experience. The clinical advisor was a family medicine physician (AC). The CoachIQ study team also collaborated with an implementation scientist with expertise in CPDs (RM) who trained the CoachIQ practice facilitators and clinical advisor on the CPD methodology and contributed to the CoachIQ program development.

**Fig. 1:**
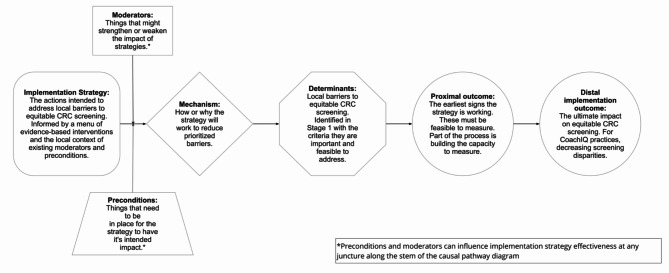
CoachIQ Causal Pathway Diagram

The CoachIQ program design was derived from creating a CPD model specific to decreasing CRC screening disparities in primary care (Fig. 1) and blending that model into standard PF approaches. The structure of the CoachIQ program was an adaptation of key elements of study team members’ (BI and AC) prior QI work around supporting PCOs in implementing system based changes to improve opioid prescribing, The Six Building Blocks, particularly the use of three facilitation stages: prepare, implement, and sustain [20–22]. The CoachIQ program incorporated an equity focus and used CPDs to inform the strategies used in three practice facilitation stages as outlined in Table 1 and detailed below.

**Table 1:** CoachIQ Program: equity and causal pathway Diagram (CPD) elements

	Equity	CPD
**Prepare** *(Months 1–4)*		
Build Team	Include representatives from targeted underserved demographics	Include representatives with knowledge of the context of CRC screening at the PCO, including existing barriers and history of QI strategies employed
Assess Baseline	Focus assessment on contextual elements, barriers, and related strategies that impact equitable CRC screening	
Prioritize Work	Prioritize high-impact barriers to equitable CRC screening and match with feasible QI strategies. Consider important preconditions, moderators, and measures	
**Implement** *(Months 5–11)*		
Implement Strategies	Implements strategies to address the prioritized barriers to equitable CRC screening	
Assess and Adjust	QI meetings include either discussions of how to track CRC screening disparities or reviews of CRC screening disparities data. When reviewing data, assess if measures are trending in the correct direction and with the intended magnitude of change. Discuss any work plan adjustments needed.	Center QI meetings on the prioritized barriers and the relationship of the existing work to the barriers. Use CPDs to identify how to overcome challenges. For example, is the precondition present? Is a moderator reducing the effect of the strategy? Discuss any work plan adjustments needed.
**Sustain** *(Month 12)*		
Assess Progress	Focus assessment on CRC screening disparities data, QI successes, and factors that might affect sustaining the implementation of strategies that reduced barriers to equitable CRC screening	
Make a Sustainability Plan	Discuss important elements of sustaining QI activities and processes that reduced barriers to equitable CRC screening.	Provide final CPDs to practices to continue the work

### Stage 1: prepare

The first stage, Prepare, occurred during the first four months of the intervention and involved building the QI team, assessing baseline, and prioritizing the work. When building their QI team, PCOs were encouraged to consider including members who represented the targeted underserved demographics, Hispanic patients, and representatives with knowledge and experience about CRC screening. To assess the baseline, the QI team completed a survey and individual members participated in interviews with the practice facilitator to assess current CRC screening practices, past improvement activities, existing barriers to screening, potential strategies to overcome those barriers, and factors that might support or impede the success of those strategies. To prioritize work, the QI team participated in a coaching meeting to discuss results of the baseline assessment and identify and prioritize barriers faced by their Hispanic patient populations, and QI improvement strategies to try that could potentially overcome those prioritized barriers. The practice facilitator used CPDs to lead the team in vetting the effectiveness of alternative strategies by assessing (1) whether strategies were clearly matched with the barriers by facilitating discussions on how the strategy would address the barrier (i.e., the mechanism), (2) whether strategies were feasible by considering the preconditions for a strategy to work and factors that could moderate how well it works, and (3) what early indicators would signal whether the strategy was working to reduce the prioritized barrier. The final product of the meeting was a CPD Action Plan outlining steps to achieve “SMART” (specific, measurable, actionable, realistic, and timebound) goals and outlining the relationships in the related CPD figures guiding the work.

### Stage 2: implement

During the seven months of the second stage, Implement, the QI team implemented the work prioritized during Stage 1 using CPD Action Plans developed at each monthly QI meeting with the practice facilitator. During monthly meetings, the facilitator used the CPD to guide the PCO in assessing their progress, reviewing early outcomes and equitable screening data, and adjusting implementation plans, as needed. A key aspect of the CoachIQ practice facilitator’s role was to interrogate whether QI team implementation activities were targeting the prioritized barriers identified during CPD assessment work in Stage 1. CoachIQ practice facilitators also aided the QI team in investigating why QI strategy implementation was struggling by checking in on the necessary strategy preconditions (e.g., clinicians available to attend the health equity training) or moderators (e.g., training materials relevant to clinician CRC screening work) that might be reducing the effectiveness of the strategy. Finally, CoachIQ practice facilitators worked with the QI team to review early outcome measures that were expected as a precursor to eventual decreasing of CRC screening disparities.

### Stage 3: sustain

During the last month of the program, the practice facilitator worked with the QI team to assess progress and what facilitated and held back the work. The practice facilitator met with the team to discuss work left to accomplish, and helped the PCO make a sustainability plan to continue the work.

### Comparison group

Throughout the study period, each PCO in the comparison group received approximately 12 months of QI support from one practice facilitator. The comparison group practice facilitator had 7 years of prior coaching experience and was not affiliated with the study. The comparison group practices had been receiving coaching on improving CRC screening for several years prior to the study start and were focused on reestablishing effective CRC screening practices and sustaining those still in effect. The QI strategies for the study period were chosen by PCOs from a list of EBIs provided by the practice facilitator. PF support involved quarterly meetings where the practice facilitator checked in on QI activities, worked through challenges, and connected the QI team to resources. There was also financial support available to these practices for staffing, patient navigation, population tracking, and patient colonoscopies.

## Results

### Characteristics

The characteristics of the CoachIQ and comparison group PF organizations are shown in Table 2. CoachIQ PCOs ranged in size from 11 to 36 primary care clinicians. The comparison group PCOs ranged in size from 19 to 74 primary care clinicians. All CoachIQ PCOs and comparison group PCOs reported using clinician reminders as an evidence-based CRC screening intervention at the start of the study. Two organizations in the comparison group and one organization in CoachIQ reported efforts to reduce structural barriers to CRC screening.

**Table 2:** Characteristics of participating Primary Care Organizations (PCOs)

	CoachIQ PCOs			Comparison Group PCOs			
	1	2	3	1	2	3	
Organization type	Health system/ hospital owned	CHC/FQHC	CHC/FQHC	CHC/FQHC	CHC/FQHC	CHC/FQHC	
Number of clinics engaged	1	2	1	8	7	9	
Number of primary care clinicians	32	11	36	74	19	33	
Primary CRC screening test preferred	Colonoscopy	None	Colonoscopy	FIT	FIT	FIT	
% of patients who are Hispanic	9%	13%	8%	6%	4%	6%	
% of all patients that report Spanish as their primary language	2%	7%	3%	7%	4%	2%	
% of patients without insurance	2%	9%	20%	1%	9%	5%	
**Evidence-based CRC screening interventions in place at baseline**							
Patient reminders	Yes	Yes	Yes	No	Yes	Yes	
Clinician assessment and feedback	No	No	Yes	No	Yes	Yes	
Clinician reminders	Yes	Yes	Yes	Yes	Yes	Yes	
Reduce structural barriers	No	No	Yes	No	Yes	Yes	

Each participating PCO reported patient characteristics for the population of patients eligible for CRC screening. In the CoachIQ PCOs, the proportion of patients identified as Hispanic ranged from 8 to 13%, and in the comparison group organizations, the range was 4–6% Hispanic. At one CoachIQ PCO, the proportion of patients without health insurance was 20%. At the remaining CoachIQ and comparison group organizations, the proportions of patients without health insurance ranged from 1 to 9%.

### Coaching

The two CoachIQ practice facilitators and the single comparison group practice facilitator entered data each month to record the coaching activities. For each activity, we calculated the proportion of months during the 12-month coaching cycle that the practice facilitator reported doing the coaching activity. CoachIQ practice facilitators reported discussing equity in the majority (75%) of monthly coaching encounters, compared to the comparison group practice facilitator who reported discussing equity in only 25% of coaching encounters (Table 3). The CoachIQ practice facilitators also reported that they facilitated prioritization of QI activities, facilitated development of an action plan, reviewed process steps and measures, and reviewed CRC screening disparities during a higher proportion of coaching encounters, compared to the comparison group practice facilitator. The comparison group practice facilitator reported providing technical support or education, connecting to others doing similar work, and sharing relevant resources at a higher proportion of coaching encounters than the CoachIQ practice facilitators.

**Table 3:** Comparison of coaching activities reported by CoachIQ practice facilitators and usual care practice facilitators

	Mean proportion of months during 12-month coaching cycle that the activity was reported by practice facilitators	
	CoachIQ	Comparison Group
Facilitated prioritization of QI activities	28%	3%
Facilitated development of an action plan	58%	8%
Discussed how the strategy is expected to affect the barrier	42%	20%
Discussed equity	75%	25%
Provided technical support or education	19%	25%
Connected to others doing similar work	3%	22%
Shared relevant resources	31%	42%
Reviewed process steps measures	42%	8%
Reviewed CRC screening disparities	44%	0%

### Examples of CPD application in CoachIQ

In addition to examining the differences reported by practice facilitators in monthly surveys about their coaching activities, we developed two case examples of how CoachIQ practice facilitators used CPDs to guide the selection and implementation of QI activities with an equity focus (see the two example CPDs in supplementary materials).

The first example involved a PCO QI team that needed to adjust their implementation approach to meet a precondition for the strategy to be effective. This PCO planned to use educational brochures to address the barriers of (1) Hispanic patients’ limited knowledge of the need for CRC screening and (2) clinicians forgetting to recommend screening. The PCO QI team theorized that the brochures would help Hispanic patients learn about the importance of CRC screening and motivate them to ask about screening during busy appointments with their primary care clinician. During a CoachIQ meeting, the QI team reported that they received the brochures and placed them in their waiting rooms. The CoachIQ practice facilitator used the CPD to prompt the team to think through whether this deployment of educational brochures would be effective. It emerged that an important precondition for the strategy to be effective might not be met. If the brochures were only in the waiting rooms, it was unclear whether the patients would notice and access them prior to their appointments. Therefore, the team adjusted their implementation approach to instead incorporate giving the brochures to patients during rooming, which would make it much more likely that the brochures would address their intended barriers and outcomes.

The CoachIQ practice facilitator also helped the QI teams use early outcome measures to confirm the strength of the strategy and barrier match. The second CPD example was for a practice using targeted patient reminders to address two prioritized barriers: (1) Patients not knowing about or forgetting about needing to be screened, (2) clinicians forgetting to recommend screening. During each CoachIQ meeting with the practice facilitator, the early outcome measure of number of Hispanic patients due for screening without a referral in the chart was monitored. This PCO made targeted outreach calls to Hispanic patients who were due for screening to encourage them to schedule an appointment and to enter information in their chart highlighting their CRC screening gap. After implementing targeted patient reminders, the number of Hispanic patients without referrals who were due for screening went from 188 in May 2023 to 16 in October 2023, serving as a strong initial indicator that the strategy was working as planned.

### Screening

**Fig. 2:**
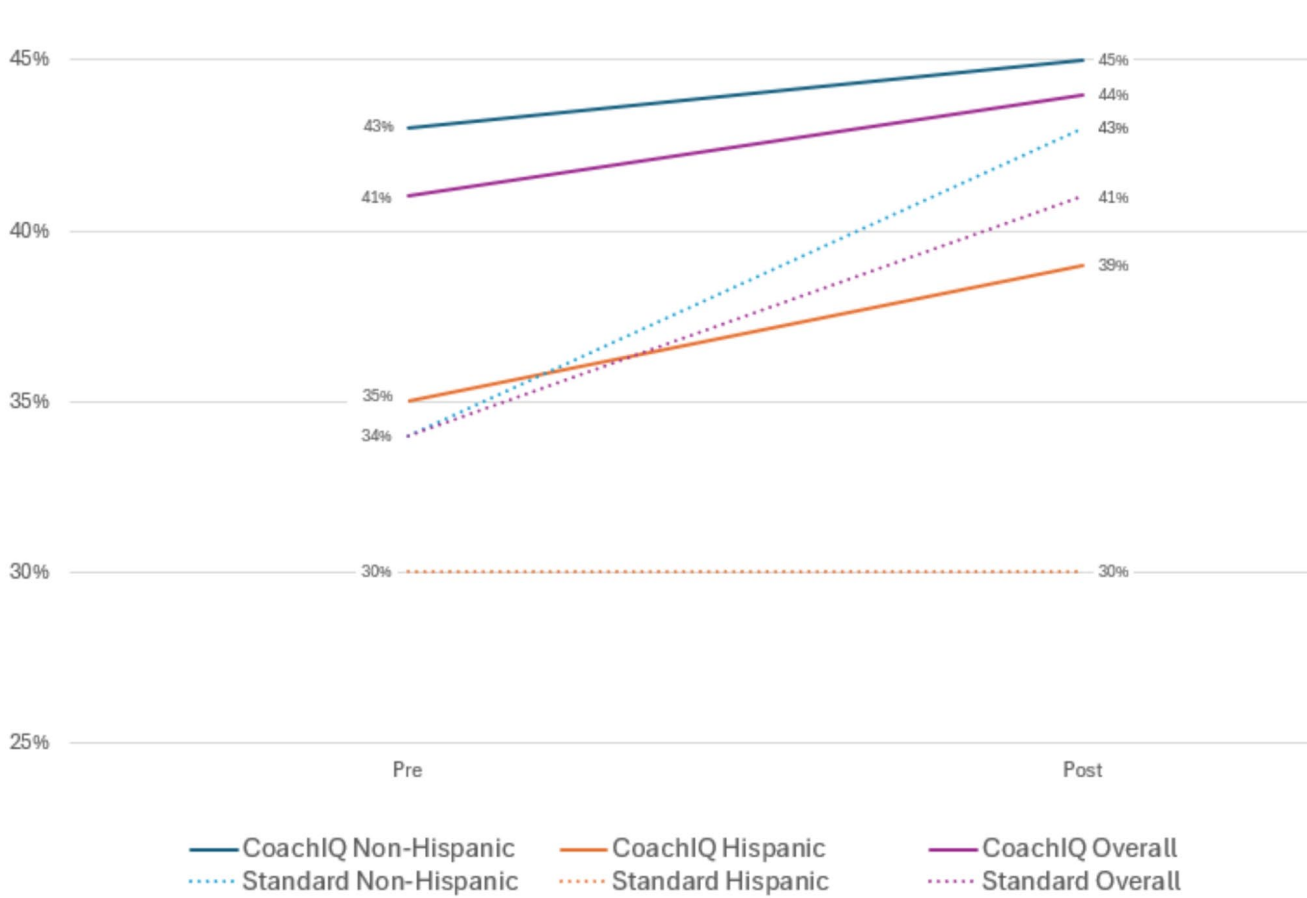
Pre-Post Colorectal Cancer Screening Rates

Figure 2 summarizes the pre/post change in the primary outcomes for the CoachIQ PCOs and the comparison group PCOs: mean CRC screening rate overall, Hispanic CRC screening rate, and non-Hispanic CRC screening rate. While the mean overall CRC screening rate in the comparison PCOs increased from 34 to 41%, the mean CRC screening rate for Hispanic patients did not increase from 30% after the period of coaching. In contrast, the mean overall CRC screening rate in the CoachIQ PCOs increased from 41 to 44%, and the mean CRC screening rate for Hispanic patients increased from 35 to 39%.

In Table 4, we report the baseline CRC screening rate, change in overall CRC screening rate, baseline CRC screening disparity, and change in CRC screening disparity for each of the CoachIQ and comparison group PCOs. The change in disparity for the CoachIQ PCOs ranged from growing by 1% in PCO 3 to reducing by 6% in PCO 1. In the comparison group, the change in disparity ranged from growing by 2% in PCO 1 to growing by 22% in PCO 3.

**Table 4:** Change in CRC screening rates and CRC screening disparities in CoachIQ and comparison group primary care organizations (PCOs)

	CoachIQ PCOs			Comparison Group PCOs		
	1	2	3	1	2	3
Baseline overall CRC screening rate	45%	41%	40%	27%	42%	32%
Change in overall CRC screening rate post intervention	+ 2%	+ 2%	+ 4%	+ 2%	0%	+ 21%
Baseline CRC screening disparity	12%	3%	8%	3%	4%	4%
Change in CRC screening disparity	-6%	-1%	+ 1%	+ 2%	+ 4%	+ 22%

## Discussion

This study designed and piloted the CoachIQ program which utilized a novel application of CPD within a PF model for decreasing CRC screening disparities. CoachIQ practice facilitators worked with PCO QI teams to prioritize barriers to equitable CRC screening and design and implement QI strategies to overcome those barriers. We demonstrate that CPD can be utilized to guide practice facilitators and PCOs in their efforts to decrease disparities in CRC screening. CPD provides an operational approach to principles for equitable QI outlined by Galifant et al., [23] including using tools for health disparity tracking and understanding contextual differences when planning implementation. The practice facilitators used CPD to help guide QI teams in selecting QI activities (i.e., strategies) that would be feasible within their context considering existing circumstances (i.e., preconditions and moderators) and those that had a clear relationship to prioritized local barriers to equitable CRC screening (i.e., outcome). The practice facilitators also took time to explore how the QI teams anticipated the activities would work to affect the barriers (i.e., mechanisms), and how to measure what the QI teams expected to see as an early result of implementation (i.e., early outcomes). Through the CoachIQ approach, the practice facilitators tracked the details of the CPDs for QI teams, prompting them through targeted questions during meetings to fine tune their QI implementation. One potential strength of the CoachIQ model is that the integration of CPD methods was accomplished with the practice facilitators, rather than direct training in the method to PCO QI teams. PCO QI teams may not have sufficient time or expertise to translate implementation science methods into actionable QI activities, [24] and the CoachIQ model provides a means to bring implementation science to PCOs without the burden of them having to identify and learn these methods.

In this study, CoachIQ practice facilitators recorded completing several activities at a greater proportion of coaching encounters compared to the comparison group practice facilitator: (1) incorporated identified barriers and prioritized activities into Action Plans for the PCOs, (2) kept equity at the forefront of coaching, and (3) consistently assessed progress to check that QI activities were having the intended effect. These activities align with the core components of CPD, suggesting practice facilitators maintained fidelity to the CoachIQ approach. Few published studies describing PF programs provide detailed data about the activities performed by practice facilitators [25]. Our approach for collecting this data was assessed as feasible by the practice facilitators and may contribute to future efforts to better characterize and compare PF approaches. Demonstrating feasible measurement and documentation of implementation strategies is a critical need in the field of implementation science [26].

QI efforts have historically failed to address, or even exacerbated health disparities [27–29]. In our study, among the three practices receiving support through CoachIQ, all three increased their CRC screening rates overall, and two practices successfully reduced CRC screening disparities for Hispanic patients. In the comparison practices, there was an increase in the Hispanic/Non-Hispanic CRC screening disparity in all three practices, despite improved overall CRC screening rates. Although we are uncertain as to why all comparison group practices increased CRC screening disparities, including a significant increase in disparities in comparison group PCO 3, we hypothesize that without intentionally focusing on equity through the QI process, there is risk for further exacerbating disparities [30]. A strength of the CoachIQ program is using both the dynamic role of the practice facilitator and a systematic approach (CPD) to potentially help the PCO engage with equity as an ongoing practice rather than a QI project finished after one cycle [23]. Improving health equity requires a systematic approach that aligns well with the CPD approach [31].

Though we did compare CRC screening outcomes for organizations receiving support from CoachIQ practice facilitators to the CRC screening outcomes for organizations receiving standard coaching through an ongoing practice facilitation program, these two groups were non-equivalent. Despite the lack of equivalency, the detailed description of the CoachIQ program and its incorporation of CPD into practice facilitation, and the demonstration that practices receiving CoachIQ support made progress in improving equitable CRC screening contributes important data on a promising implementation science approach to decreasing CRC screening disparities. The organizations in the two arms received different financial incentives, which is a potential cofounder of the effect observed. For the pilot study, CoachIQ teams were encouraged to include Hispanic patients. Future versions of the program could go farther and include patients more intentionally as part of the baseline assessment process, and throughout implementation.

## Conclusion

The CoachIQ program merges two implementation science methodologies, PF and CPD, to help PCOs focus QI efforts on improving CRC screening while also reducing screening disparities. Results from this pilot study demonstrate key differences between CoachIQ facilitation and standard facilitation, and point to the potential of the CoachIQ approach in decreasing disparities in CRC screening.

### Electronic supplementary material

Below is the link to the electronic supplementary material.

Supplementary Material 1: Additional File 1: CoachIQ Causal Pathway Diagram Example 1. Description of data: A diagram outlining the first case study example of applying the Causal Pathway Diagram in the CoachIQ program.

Supplementary Material 2: Additional File 2: CoachIQ Causal Pathway Diagram Example 2. Description of data: A diagram outlining the second case study example of applying the Causal Pathway Diagram in the CoachIQ program.

Supplementary Material 3: Additional File 3: CoachIQ Practice Facilitator Monthly Survey. Description of data: A monthly electronic survey practice facilitators completed about coaching activities conducted with each primary care organization during the prior month.

## Data Availability

The datasets used and/or analyzed during the current study are available from the corresponding author on reasonable request.

## Abbreviations

CoachIQ: Coaching to Improve Colorectal Cancer Screening Equity
CPD: Causal Pathway Diagram
CRC: Colorectal Cancer
EBI: Evidence Based Intervention
PCO: Primary Care Organization
PF: Practice Facilitation
QI: Quality Improvement
US: United States
