# Supplementary figures and images for: Integrating causal pathway diagrams into practice facilitation to address colorectal cancer screening disparities in primary care
Source: BMC Health Serv Res. 2024 Aug 30;24:1007. doi: 10.1186/s12913-024-11471-5 (PMC11365243; doi:10.1186/s12913-024-11471-5)

**Additional File 1: CoachIQ Causal Pathway Diagram Example 1**


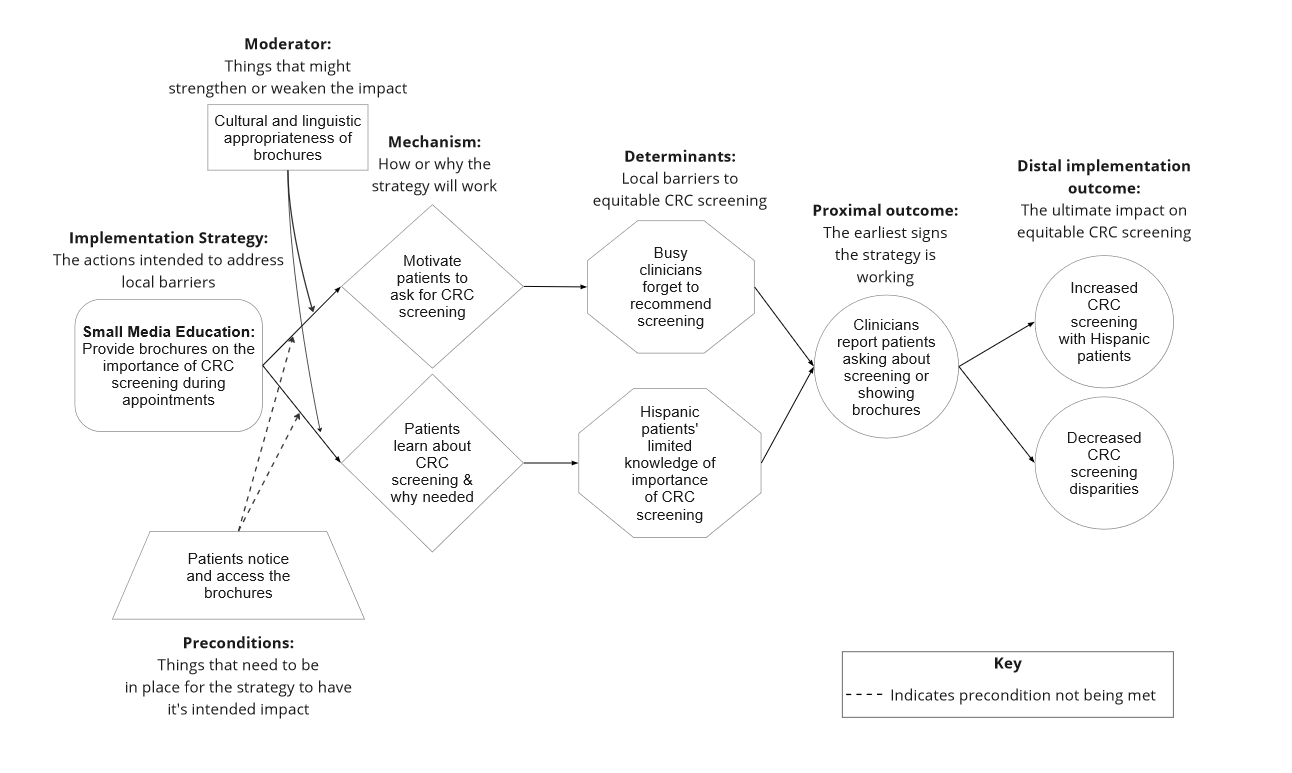

Supplement: Supplementary file 1 — Supplementary Material 1: Additional File 1: CoachIQ Causal Pathway Diagram Example 1. Description of data: A diagram outlining the first case study example of applying the Causal Pathway Diagram in the CoachIQ program. [file 12913_2024_11471_MOESM1_ESM.docx]

**Additional File 2: CoachIQ Causal Pathway Diagram Example 2**


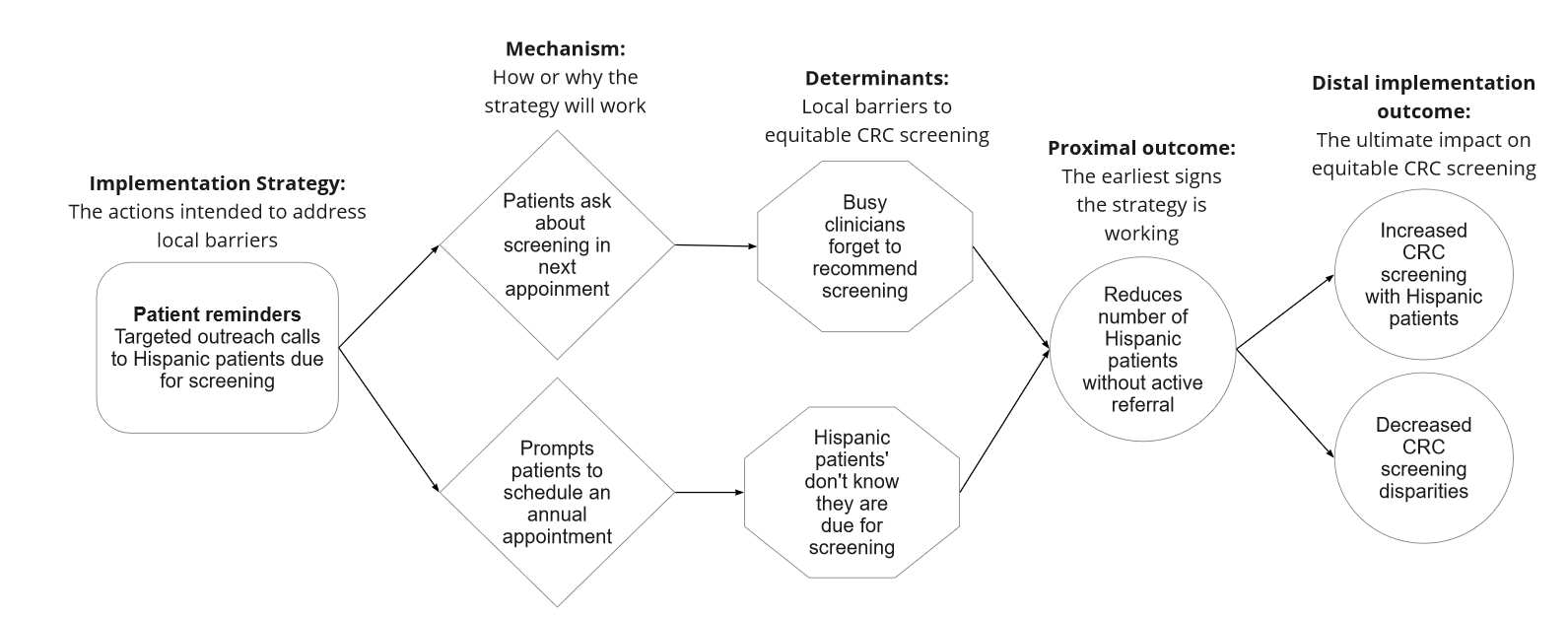

Supplement: Supplementary file 2 — Supplementary Material 2: Additional File 2: CoachIQ Causal Pathway Diagram Example 2. Description of data: A diagram outlining the second case study example of applying the Causal Pathway Diagram in the CoachIQ program. [file 12913_2024_11471_MOESM2_ESM.docx]
