# Supplementary material for: Integrating causal pathway diagrams into practice facilitation to address colorectal cancer screening disparities in primary care
Source: BMC Health Serv Res. 2024 Aug 30;24:1007. doi: 10.1186/s12913-024-11471-5 (PMC11365243; doi:10.1186/s12913-024-11471-5)
Supplement: Supplementary file 3 — Supplementary Material 3: Additional File 3: CoachIQ Practice Facilitator Monthly Survey. Description of data: A monthly electronic survey practice facilitators completed about coaching activities conducted with each primary care organization during the prior month. [file 12913_2024_11471_MOESM3_ESM.docx]

**Additional File 3: CoachIQ Practice Facilitator Monthly Survey**

*The following questions will be sent in an electronic survey to be completed once a month for the prior month’s work. You will complete one survey per month for each primary care organization. Thank you!*

1. How did you interact with the organization this month?
   - Quality improvement meeting
     - Duration of contact:
     - Number of organization attendees:
   - Shared learning call
   - Email
   - Phone
   - Text
   - Other
   - None
2. Was a quality improvement meeting cancelled? y/n
   - If yes, why?
3. Were there any disruptions at the organization? y/n
   - If yes, what?
4. What, if any, colorectal cancer screening barrier/facilitator work did you do with the organization this month?
   - Assessed what barriers/facilitators exist
   - Prioritized barriers/facilitators
     - If yes, what were the prioritized barriers/facilitators?
   - Discussed strategies to overcome barriers/facilitators
   - Other
   - None of the above
5. What, if any, quality improvement implementation support work did you do with the organization this month?
   - Assessed what current quality improvement activities exist
   - Matched quality improvement activities to barriers
   - Discussed what is needed for a quality improvement activity to work (moderators and preconditions)
   - Discussed how a quality improvement activity (strategy) is expected to affect a barrier (mechanism)
   - Assessed progress of quality improvement activity implementation
   - Discussed how to overcome challenges in quality improvement activity implementation
   - Celebrated successes of quality improvement activity implementation
   - Facilitated prioritization of quality improvement activities for the coming month
     - If yes, what were the prioritized activities?
   - Facilitated development of a plan for the quality improvement activities for the coming month (steps, responsible parties, timeline)
   - Discussed equity
   - Provided technical support or education
   - Connected to others doing similar work
   - Shared relevant resources
   - Other
   - None
6. What, if any, measuring success support work did you do with the organization this month?
   - Set measures of success
     - If yes, what were the measures of success?
   - Provided technical support or education to help identify how to measure success using existing resources
   - Connected to others doing similar work
   - Shared relevant resources
   - Reviewed barriers/facilitators measures
   - Reviewed process steps measures
   - Reviewed measures of the mechanism by which the quality improvement activity should affect the barrier/facilitator
   - Reviewed early outcome measures
   - Reviewed colorectal cancer screening rate
   - Reviewed colorectal cancer screening disparities between Hispanic and non-Hispanic patients
   - Other
   - None
7. Other coaching work done this month:
8. If known, provide a brief description of the work the clinic did this month:
